# Genome Sequence of *Klebsiella pneumoniae* Strain LCT-KP182, Which Acquired Hemolytic Properties after Space Flight

**DOI:** 10.1128/genomeA.01088-13

**Published:** 2014-01-09

**Authors:** Xiaojun Zhang, Tong Wang, Wenbin Liu, Yinghua Guo, Junfeng Wang, Tianzhi Li, Xiangqun Fang, Xuelin Zhang, Wenkui Dai, Changting Liu

**Affiliations:** Nanlou Respiratory Diseases Department, Chinese PLA General Hospital, Beijing, Chinaa; BGI-Shenzhen, Shenzhen, Chinab

## Abstract

The *Klebsiella pneumoniae* strain LCT-KP182 acquired hemolytic properties after space flight. Here, we present the draft genome sequence of this strain.

## GENOME ANNOUNCEMENT

It has been confirmed that there are effects on microbial characteristics that contain arrangement of the antibiotic sensitive test, virulence, and biochemical metabolism. *Klebsiella pneumoniae*, which originated from the China General Microbiological Culture Collection Center (CGMCC) as strain CGMCC 1.1736, was carried by the ShenZhouVIII spacecraft for 398 h. After space flight, we found a *K. pneumoniae* strain named LCT-KP182, with hemolytic properties (1).

Whole-genome sequencing was performed using Illumina HiSeq 2000 (Illumina, Inc., USA) by generating paired-end libraries (500 bp and 6 kb) according to the manufacturer’s instructions. From these libraries, 720 Mb and 356 Mb of high-quality data were generated, respectively, with a read length of 90 bp. The paired-end reads were assembled using SOAP*denovo* version 1.05. In order to verify the plasmids, we used the assembly result to align with the NCBI plasmid database. The coding sequences (CDSs) were predicted by using Glimmer version 3.02. All the genes were subjected to homologous comparison by BLAST with the NCBI nonredundant public database and other databases, such as KEGG, COG, and Swiss-Prot for function annotation. rRNAs and tRNAs were identified using RNAmmer and tRNAscan-SE 1.21, respectively. Meanwhile, tandem repeats were predicted using the TandemRepeatFinder (TRF) software.

Finally, we obtained 7 scaffolds consisting of 23 contigs with a total length of 5,801,744 bp, and the G+C content was determined to be 56.93%. Nearly 10.84% of the sequence aligned well with the plasmid database. From the genome composition analysis results, we found that the genome contains 5,552 genes, with an average length of 905 bp, and the total length of genes is 5,024,301 bp, which makes up 86.60% of the genome. Among the whole-gene sets, 3,945 CDSs were involved in 22 functional COG groups, and 3,654 CDSs are involved in 33 metabolic pathway KEGG groups. We found 87 tRNAs with a total length of 6,817 bp, which makes up 0.1174% of the genome. In addition, 26 rRNAs and 39 small RNAs (sRNAs) were also determined. Furthermore, 119 tandem repeats, 53 minisatellite DNAs, and 12 microsatellite DNAs were found in the genome.

### Nucleotide sequence accession number.

This whole-genome shotgun project of *K. pneumoniae* LCT-KP182 has been deposited at DDBJ/EMBL/GenBank under the accession no. ATRN00000000. The version described in this paper is the first version.
